# Book Notices

**Published:** 1892-03

**Authors:** 


### Book Notices.

INTERNATIONAL CLINICS: A Quarterly of Clinical Lectures on Medicine, Surgery, Gynecology, Pediatrics, Neurology, Dermatology, Ophthalmology and Otology. By Professors and Lecturers of the leading Medical Colleges of the United States, Great Britain and Canada: Edited by Jno. M. Kealing, M. D., Philadelphia; J. P. Crozer Griffith, M. D., Philadelphia; J. Mitchell Bruce, M. D., F. R. C. P., London, England; David W. Finlay, M. D., F. R. C. P., London, England.

These books are well bound, and contain much of value to medical men. Volumes for April and October have been received at this office. Each contains about 300 pages. B.

INSOMNIA AND ITS THERAPEUTICS. By A. W. Macfarlane, M. D., Fellow of the Royal Medical and Chirurgical Society of London; Examiner in Medical Jurisprudence in the University of Glasgow; Honorary Consulting Physician (late Physician) Kilmarnock Infirmary; Formerly Examiner in Medicine and Clinical Medicine in the University of Glasgow, etc., etc. (Reprinted from Wood's Medical and Surgical Monographs.) Octavo, 302 pages, muslin, $175. New York, William Wood & Company.

The above work is regarded as one of the best treatises published on insomnia. B.

THE MOTHER'S HAND BOOK.—A practical treatise on the management of children in health and disease; with an appendix, containing articles on diseases and accidents that may suddenly happen to grown persons. By Levin J. Woollen, M. D. 419 pages. Richmond, Va.: Everett Waddey Co., Publishers and Printers: 1891.

MODERN MEDICAMENTS.—Before us is the latest list, just issued, of the extensive line of standard and pharmaceutical preparations, and modern medicaments manufactured by Parke, Davis & Co.

A novel and attractive feature of their comprehensive and well arranged list is the representation, by forty engravings, of the home laboratories at Detroit, and the branches at New York, Kansas City, and Walkerville, Ontario. These engravings comprise views of the exterior and interior of the laboratories, offices, and sections of departments at Detroit. The cover is engraved, and altogether it is the most complete list ever published of the products of this well-known house, and will be welcomed by their friends. To physicians interested a copy ot this list will be mailed on request. Mention this journal.

---

"EMINENT AMERICAN PHYSICIANS AND SURGEONS.—Carlon & Hollenbeck, Indianapolis, Ind., have issued a prospectus of a work under the above title, to be edited by Dr. R. French Stone. The work will be sold by subscription only, and it is expected that it will be issued before the end of the year. An edition of 5,000 to 10,000 will be issued, according to the number of subscribers secured, while the book is in preparation. For terms, blanks and information address the publishers or Dr. Stone, Editor and Business Manager, 16 West Ohio st., Indianapolis, Ind.

